# Comparison of scales for the evaluation of aneurysmal subarachnoid haemorrhage: a retrospective cohort study

**DOI:** 10.1007/s00330-024-10814-4

**Published:** 2024-06-05

**Authors:** David Couret, Salah Boussen, Dan Cardoso, Audrey Alonzo, Sylvain Madec, Anthony Reyre, Hervé Brunel, Nadine Girard, Thomas Graillon, Henry Dufour, Nicolas Bruder, Mohamed Boucekine, Olivier Meilhac, Pierre Simeone, Lionel Velly

**Affiliations:** 1 Department of Anaesthesiology and Critical Care Medicine, Aix Marseille Univ, University Hospital Timone, Marseille, France; 2 Neurocritical Care Unit, University Hospital Saint Pierre, Réunion Univ, BP 350, Saint Pierre, 97448 La Réunion France; 3 Reunion Island University, INSERM, Diabète Athérothrombose Réunion Océan Indien (DéTROI), Saint Denis de la Réunion, France; 4 Department of Radiology, University Hospital Timone, Aix Marseille University, Marseille, France; 5 Department of Neurosurgery, University Hospital Timone, Aix Marseille University, Marseille, France; 6 Centre D’Etudes Et de Recherches Sur Les Services de Santé Et Qualité, Faculté de Médecine, Aix-Marseille Université, 13005 Marseille, France; 7 CNRS, INT, Inst Neurosci Timone, Aix Marseille Univ, Marseille, France

**Keywords:** Fisher scale, Hijdra scale, Subarachnoid haemorrhage, Delayed cerebral infarct, Vasospasm

## Abstract

****Background/**Objectives:**

Aneurysmal subarachnoid haemorrhage (aSAH) is a life-threatening event with major complications. Delayed cerebral infarct (DCI) occurs most frequently 7 days after aSAH and can last for a prolonged period. To determine the most predictive radiological scales in grading subarachnoid or ventricular haemorrhage or both for functional outcome at 3 months in a large aSAH population, we conducted a single-centre retrospective study.

**Methods:**

A 3-year single-centre retrospective cohort study of 230 patients hospitalised for aSAH was analysed. Initial computed tomography (CT) scans in patients hospitalised for aSAH were blindly assessed using eight grading systems: the Fisher grade, modified Fisher grade, Barrow Neurological Institute scale, Hijdra scale, Intraventricular Haemorrhage (IVH) score, Graeb score and LeRoux score.

**Results:**

Of 200 patients with aSAH who survived to day 7 and were included for DCI analysis, 39% of cases were complicated with DCI. The Hijdra scale was the best predictor for DCI, with a receiver operating characteristic area under the curve (ROC_AUC_) of 0.80 (95% confidence interval (CI), 0.74–0.85). The IVH score was the most effective grading system for predicting acute hydrocephalus, with a ROC_AUC_ of 0.85 (95% CI, 0.79–0.89). In multivariate analysis, the Hijdra scale was the best predictor of the occurrence of DCI (hazard ratio, 1.18; 95% CI, 1.10–1.25).

**Conclusions:**

Although these results have yet to be prospectively confirmed, our findings suggest that the Hijdra scale may be a good predictor of DCI and could be useful in daily clinical practice.

**Clinical relevance statement:**

Better assessment of subarachnoid haemorrhage patients would allow for better prognostication and management of expectations, as well as referral for appropriate services and helping to appropriate use limited critical care resources.

**Key Points:**

- *Aneurysmal subarachnoid haemorrhage is a life-threatening event that causes severe disability and leads to major complications such as delayed cerebral infarction.*
- *Accurate assessment of the amount of blood in the subarachnoid spaces on computed tomography with the Hijdra scale can better predict the risk of delayed cerebral infarct.*
- *The Hijdra scale could be a good triage tool for subarachnoid haemorrhage patients.*

## Introduction

Aneurysmal subarachnoid haemorrhage (aSAH) is a life-threatening event. Severe complications can occur after the aneurysm is secured, such as delayed cerebral infarct (DCI) or acute hydrocephalus requiring intensive care monitoring for 12–21 days after aSAH [1, 2]. The management of these complications in high-volume hospitals with neurosurgical and endovascular services seems to be associated with better outcomes [3]. However, specialised hospitals have limited capacity in terms of neurocritical care unit (NCCU) beds. Optimising resource allocation requires the ability to select patients at high risk of complications after aSAH. In this context, a radiological score that would be predictive of complications would be useful for identifying patients who need intensive care unit (ICU) monitoring.

Since the early 1980s and the publication of the Fisher grade, the occurrence of vasospasm and prognosis with aSAH has been recognised as being influenced by the severity of the initial bleeding, which can be evaluated on an early computed tomography (CT) scan [4–12]. However, several studies demonstrated low sensitivity and specificity of this scale for predicting DCI [6–10]. Recently, a systematic review assessing the association of radiological scales for grading aSAH with DCI showed that patients with Fisher grade 4 have a significantly lower risk of DCI compared to those with Fisher grade 3 [13]. This is probably due to the Fisher grading system. In fact, Fisher grade 3 is for diffuse thick SAH, and Fisher grade 4 is for intraventricular haemorrhage (IVH) or intracerebral haemorrhage (ICH) and diffuse SAH or not. With current clinical management including nimodipine, hypertensive therapy and endovascular treatment, the Fisher grade predicts symptomatic vasospasm in only half of cases [14]. For this reason, other radiological scales have been developed to assess the amount of blood present in the subarachnoid spaces [7–10]. These scales qualitatively divide aSAH into categories, as do the Claassen scale [7] and the modified Fisher grade [9], or involve a semi-quantitative assessment, as do the Hijdra [8] or the Barrow Neurological Institute (BNI) [10] grading scales. Other grading systems, including the Graeb [15] or LeRoux scales [16], or the Intraventricular Haemorrhage (IVH) score [17], use a semi-quantitative method to assess the amount of blood present in the ventricles.

The aim of our study is to evaluate the predictive performance of eight radiological scales used in aSAH for the occurrence of DCI, acute hydrocephalus and functional outcome at 3 months to enable better triage of patients and provide them with a care offer adapted to their severity.

## Methods

Guidelines for reporting this study were derived from the “STrengthening the Reporting of Observational Studies in Epidemiology” (STROBE) Statement [18].

### Study design and population

This was a single-centre retrospective cohort study of consecutive patients with an aSAH admitted during a 33-month period (January 1, 2013 to July 30, 2016) at our neurocritical care unit (NCCU). Access to health information was approved by an ethics committee (Comité d’éthique pour la recherche en Anesthésie‐Réanimation—IRB 000102542019081), which waived the requirement for individual consent according to French law at the time of the study [19]. Inclusion criteria were age older than 18 years, an available head CT scan demonstrating aSAH prior to any neurosurgical intervention (external ventricular drainage (EVD), aneurysm clipping or endovascular treatment), and confirmed ruptured aneurysm on subsequent digital subtraction angiography. Exclusion criteria were the presence of non-aneurysmal vascular malformations and intracranial artefacts (prior embolisation or aneurysm clipping) for outcome evaluation and death before day 7 after admission for DCI evaluation. Patients with SAH from other causes such as head injury, arteriovenous malformation, or arterial dissection or without aneurysms confirmed on CT or angiography were excluded. Patients for whom an initial CT could not be retrieved, with incomplete CT, or with an initial CT obtained more than 24 h after bleeding were also excluded.

### Clinical management

Our aSAH management policy has been described previously in detail [1]. Briefly, all patients were managed in a dedicated NCCU according to a standardised aSAH protocol in accordance with published European guidelines [20]: administration of intravenous nimodipine and ventricular drainage in cases of hydrocephalus allowing continuous monitoring of intracranial pressure. All aneurysms were secured within 24 h after admission with endovascular coil embolisation or surgery. All patients benefit from follow-up brain imaging within 24–48 h post aneurysm securing procedure. Subsequently, brain scans are performed in the event of neurological deterioration or to monitor EVD weaning tests. All patients were followed with transcranial Doppler sonography (vasospasm was suspected when flow velocity reached 120 cm/s in the middle cerebral artery, or when a rapid increase in flow velocity was observed [21]). Only the modified Fisher grade was used routinely in our institution. Those with neurological symptoms deemed suspicious for vasospasm underwent CT angiography, followed by conventional angiography in case of moderate or severe vasospasm.

### CT grading

**Fig. 1:**
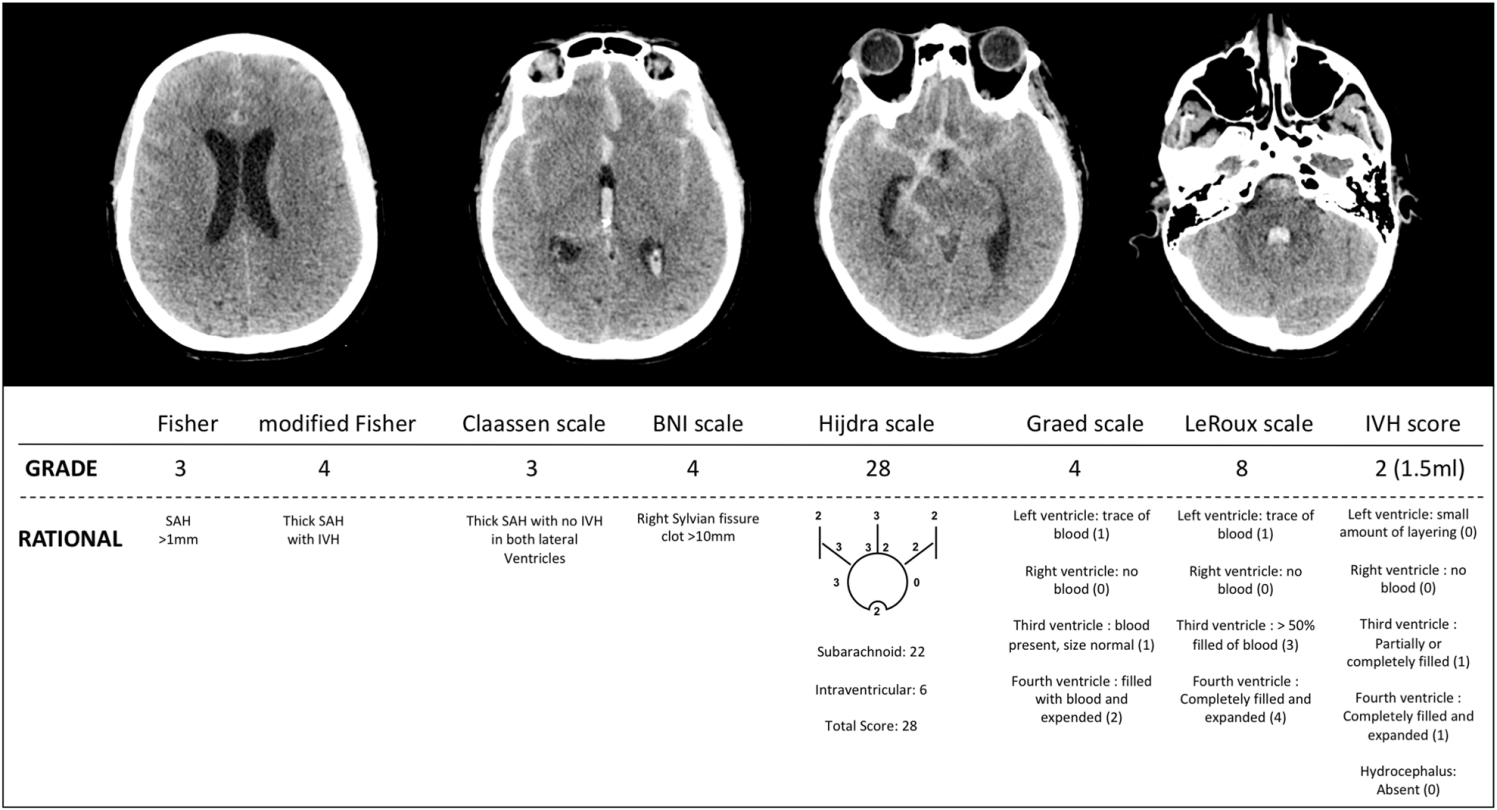
Illustration of the result of each of the eight CT scan-based grading systems from patient 6 h after subarachnoid haemorrhage from anterior communicating artery aneurysm

For each patient, two independent clinicians blinded to clinical data retrospectively reviewed the initial 32-slice CT scans. Each head CT was graded after patient hospitalisation according to eight grading systems: the Fisher grade [4], modified Fisher grade [9], Claassen scale [7], Hijdra scale [8], BNI scale [10], Graeb scale [15], LeRoux scale [16] and IVH score [17]. Each grading system is detailed in Additional file 1 (Table S1), and an illustrative example of patient evaluation is depicted in Fig. 1.

### Data collection, DCI definition and outcome assessment

Data corresponding to clinical characteristics such as age, sex, Glasgow Coma Scale (GCS) [22] and World Federation of Neurosurgical Societies (WFNS) scale [23] were recorded at admission. The Simplified Acute Physiology Score 2 (SAPS II) [24] was calculated within 24 h after NCCU admission. Specific neurologic events (intracerebral haematoma, rebleeding, neurogenic pulmonary oedema, seizure and cerebral salt-wasting syndrome (CSWS)) were collected. In our institution, the definition of CSWS is the most widely used in the literature, namely hyponatremia with elevated urinary sodium and hypovolemia.

Based on the latest recommendations [25, 26], DCI was defined as follows: development of focal neurologic signs; reduction by at least two points on the GCS that lasts for at least 1 h and is associated with angiographic cerebral vasospasm, detected either with CT angiography or digital subtraction angiography (severe arterial narrowing on digital subtraction angiography not attributable to atherosclerosis, catheter-induced spasm, or vessel hypoplasia as determined by our neuroradiologist); or a new cerebral infarction detected on a CT scan, either within 6 weeks after an aSAH or before discharge, after excluding a procedure-related infarction.

Acute hydrocephalus was defined as a build-up of cerebrospinal fluid and as the need to place an EVD within the first 72 h. It was diagnosed when the bicaudate index was greater than the 95th percentile for age on a CT scan within 72 h of the ictus [27]. Rebleeding was defined by either an acute deterioration in neurologic status in conjunction with a new haemorrhage apparent on a CT scan or an increase in haemorrhage burden on a repeat CT scan.

The outcome was evaluated at 3 months after the bleeding using the Glasgow Outcome Scale Extended (GOSE) and dichotomised into poor (GOSE 1–4) and good outcomes (GOSE 5–8) [28].

### Statistical analysis

Means and standard deviations were calculated for continuous variables with normal distributions, and medians and interquartile ranges for non-parametric variables. For categorical variables, numbers and percentages were used. Comparison between continuous variables from two groups was assessed by an unpaired two-sample *t*-test (normally distributed) or a Wilcoxon–Mann–Whitney *U*-test (no assumption for distribution). Differences between categorical variables were assessed by Fisher’s exact test. A receiver operating characteristic (ROC) curve was plotted to determine the ROC area under the curve (ROC_AUC_) and the optimal cut-off value of grading scales that best predicted DCI, early hydrocephalus requiring EVD and poor outcome. The ROC_AUC_ of each scale was compared with those with higher ROC_AUC_ values, using the method described by DeLong et al [29]. The interobserver variability of the eight scales was assessed. A weighted Cohen kappa coefficient (*κ*) was calculated for each pair per scale used with *κ* < 0.2, *κ* = 0.21 to 0.4, *κ* = 0.41 to 0.6, *κ* = 0.61 to 0.8, and *κ* > 0.8 corresponding to poor, fair, moderate, strong, and near-complete agreement, respectively [30]. A multiple logistic regression analysis was also performed to assess the risk factors for DCI, early hydrocephalus requiring EVD, and poor outcome. Odds ratios in this study indicate those for 1-SD changes of explanatory variables. All variables with probability values < 0.20 in the univariate analysis were then candidates in the multivariate analysis with a stepwise forward selection of the variables. In the final models, variables with probability values < 0.05 were considered significant. The results were expressed as odds ratios with 95% confidence intervals (CIs). All statistics were carried out using JMP (version 14.0, SAS) except for the ROC analyses and kappa coefficient calculation, which were performed using MedCalc (version 9.2, MedCalc Software). ICC estimates and their 95% CIs were calculated using IBM SPSS statistical package version 19 (SPSS Inc) based on a single-rating, absolute-agreement, two-way mixed-effects model. Statistical significance was assumed at *p* values of 0.05 and below.

## Results

### Patient demographics

**Fig. 2:**
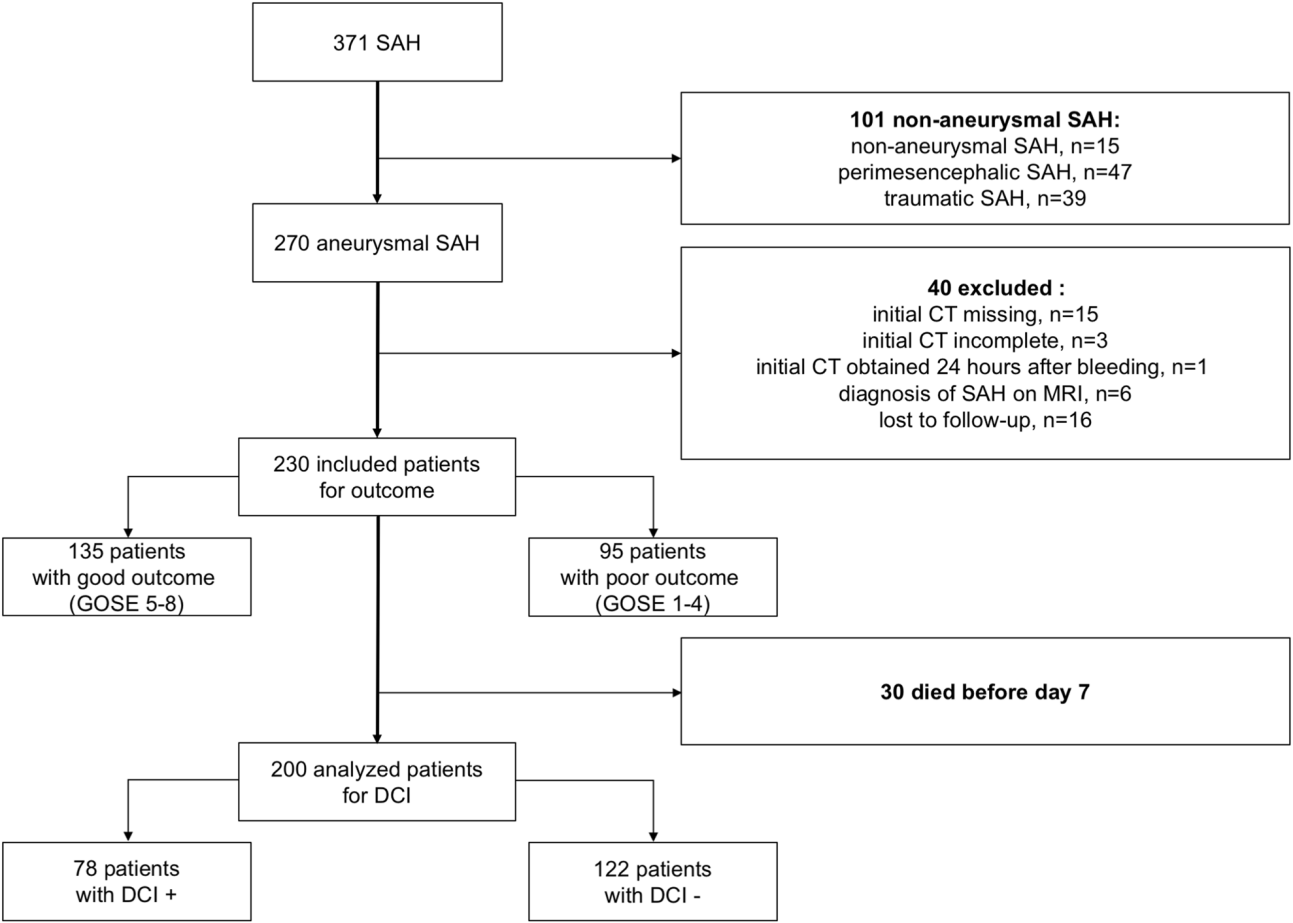
Flow of patients with subarachnoid haemorrhage (SAH) within this cohort. CT, computerised tomography; DCI, delayed cerebral infarct; GOSE, Glasgow outcome scale extended; MRI, magnetic resonance imaging

During the study period, of 371 consecutive patients with SAH, 270 suffered acute aSAH and 230 met all inclusion criteria for outcome evaluation (Fig. 2). Of these, 200 patients survived for more than 7 days and were included in the analysis for factors related to DCI. Patient characteristics are presented in Table 1. A total of 48% of patients were classified as grade 3–5 on the WFNS scale. The in-hospital mortality rate was 24%. DCI was documented in 78 patients (39%) and was related to poor outcomes (GOSE with DCI: 6 (3–8) vs. without DCI: 8 (4–8); *p* = 0.02). Other variables are related to DCI occurrence such as GCS at admission (GCS with DCI: 13 (7–14) vs. without DCI: 14 (10–15); *p* = 0.01), WFNS scale at admission (WFNS with DCI: 3 (2–4) vs. without DCI: 2 (1–4); *p* = 0.01), neurogenic pulmonary oedema (with DCI: 8 (10) vs. without DCI: 2 (2); *p* = 0.01) and angiographic vasospasm (with DCI: 78 (99) vs. without DCI: 5 (4); *p* < 0.001). Poor neurological condition (WFNS 3–5) at admission to NCCU was statistically associated with poor neurological outcome (*p* < 0.001) and occurrence of DCI (*p* = 0.01). Other variables were associated with poor outcomes (middle cerebral artery localisation of ruptured aneurysm *p* = 0.01; posterior communicant artery *p* = 0.04; and early complications like hydrocephalus *p* = 0.003, intracerebral haematoma *p* < 0.001 and rebleeding *p* < 0.001). Early hydrocephalus requiring EVD occurred in 121 (53%) of all patients included in the global analysis. Univariate analysis for factors associated with death showed IGS2 *p* < 0.001 and rebleed *p* = 0.005 (Additional file 1—Table S2).

**Table 1:** Baseline characteristics of patients with aneurysmal subarachnoid haemorrhage that developed poor or good outcomes and that developed or did not delay cerebral ischaemia (DCI)

Variable	Outcome (*n* = 230)				DCI^a^ (*n* = 200)		
	All patients (*n* = 230)	Good (GOSE 5-8) (*n* = 135)	Poor (GOSE 1-4) (*n* = 95)	*p* value^b^	No (*n* = 122)	Yes (*n* = 78)	*p* value^c^
Demographic characteristics							
Male sex—no. (%)	96 (42)	49 (36)	47 (49)	0.47	47 (39)	33 (42)	0.66
Age—years	54 ± 13	52 ± 12	56 ± 13	**0.01**	54 ± 13	52 ± 12	0.20
Clinical presentation							
GCS	13 (7–15)	14 (13–15)	8 (5–12)	**< 0.001**	14 (10–15)	13 (7–14)	**0.01**
SAPS II	34 (21–49)	25 (18–34)	47 (39–54)	**< 0.001**	28 (20–41)	33 (20–47)	0.15
WFNS scale	3 (1–4)	2 (1–4)	4 (3–5)	**< 0.001**	2 (1–4)	3 (2–4)	**0.01**
3–5—no. (%)	107 (47)	37 (27)	70 (74)	**< 0.001**	51 (42)	45 (58)	**0.03**
Ruptured aneurysm location—no. (%)							
Anterior circulation	186 (81)	106 (79)	80 (84)	0.31	100 (82)	63 (81)	0.85
ICA	38 (17)	27 (20)	11 (12)	0.11	20 (16)	12 (15)	1.00
ACA	20 (9)	16 (12)	4 (4)	0.06	14 (11)	5 (6)	0.32
MCA	48 (21)	20 (15)	28 (29)	**0.01**	29 (24)	15 (19)	0.49
AcomA	80 (35)	43 (32)	37 (39)	0.33	37 (30)	31 (40)	0.22
Posterior circulation	44 (19)	29 (21)	15 (16)	0.31	22 (18)	15 (19)	0.85
AcomP	17 (7)	14 (10)	3 (3)	**0.04**	10 (8)	7 (9)	1.00
ACP	3 (1)	1 (1)	2 (2)	0.57	1 (1)	2 (3)	0.56
PICA/AICA/SCA	5 (2)	3 (2)	2 (2)	1.00	3 (3)	1 (1)	1.00
BA	16 (7)	10 (7)	6 (6)	0.80	6 (5)	5 (6)	0.75
VA	3 (1)	1 (1)	2 (2)	0.57	2 (2)	0 (0)	0.52
Aneurysm treatment—no. (%)							
Coiling/clipping	164 (71)/66 (29)	114 (84)/21 (16)	50 (53)/45 (47)	**< 0.001**	91 (75)/31 (25)	64 (82)/14 (18)	0.23
Complications—no. (%)							
Early hydrocephalus requiring EVD	121 (53)	60 (44)	61 (64)	**0.003**	57 (47)	48 (62)	0.38
Intracerebral haematoma	69 (30)	24 (18)	45 (47)	**< 0.001**	36 (30)	20 (26)	0.63
Rebleeding	21 (9)	5 (4)	16 (17)	**< 0.001**	11 (9)	6 (8)	0.52
Neurogenic pulmonary oedema	10 (4)	7 (5)	3 (3)	0.53	2 (2)	8 (10)	**0.01**
Angiographic vasospasm	81 (35)	48 (36)	33 (35)	1.00	5 (4)	78 (99)	**< 0.001**
Minor	30 (13)	19 (14)	11 (12)	0.69	5 (4)	23 (29)	**< 0.001**
Moderate	28 (34)	19 (39)	9 (26)	0.31	0 (0)	28 (36)	**< 0.001**
Severe	26 (31)	11 (22)	15 (44)	0.09	0 (0)	26 (33)	**< 0.001**
Cerebral salt-wasting syndrome	12 (5)	6 (4)	6 (6)	0.56	6 (5)	6 (8)	0.41
Seizure	14 (6)	9 (7)	5 (5)	0.78	8 (7)	5 (6)	1.00
Outcomes							
GOSE at 3 months after SAH	7 (2–8)	8 (7,8)	1 (1–3)	**< 0.001**	8 (4–8)	6 (3–8)	**0.02**
In-hospital mortality—no. (%)	55 (24)	0 (0)	55 (58)	**< 0.001**	16 (13)	9 (12)	0.83

Results are expressed as numbers (%), medians (interquartile range), or means ± SD

*ACA* anterior cerebral artery, *ACP* posterior cerebral artery, *AICA* anterior inferior cerebellar artery, *AcomA* anterior communicating artery, *AcomP* posterior communicating artery, *BA* basilar artery, *EVD* external ventricular drain, *GCS* Glasgow Coma Scale, *GOSE* Glasgow Outcome Scale Extended, *ICA* internal carotid artery, *MCA* middle cerebral artery, *SAPS II* Simplified Acute Physiologic Score II, *SCA* superior cerebellar artery, *PICA* posterior inferior cerebellar artery, *VA* vertebral artery, *WFNS* World Federation of Neurological Surgeons Grading System

^a^ Data for DCI were collected from a collective of 200 patients (30 patients who died before day 7 were excluded)

^b^ *p* value for patients with good outcomes vs. poor outcomes

^c^ *p* value for patients with DCI vs. without DCI. Boldface values represent significant findings assumed at *p* values of 0.05 and below

### Predictive value of CT grading systems

**Fig. 3:**
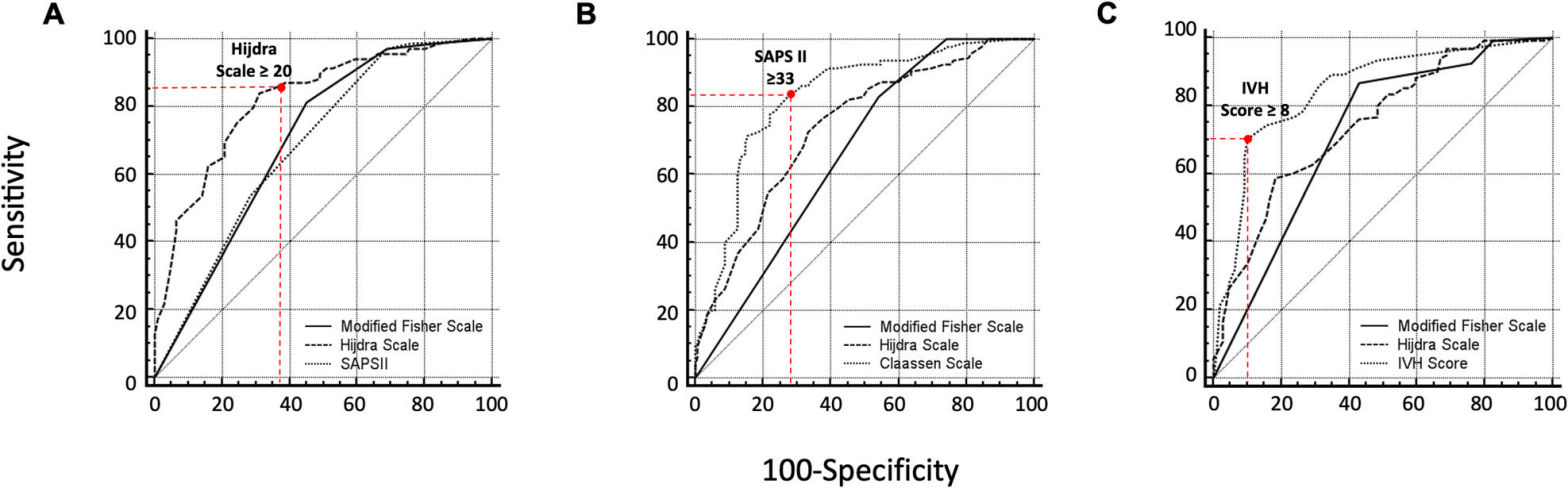
ROC curves for the different qualitative, semi-quantitative and quantitative measures used for determining the development of delayed cerebral infarct (**A**), poor outcome (**B**) and early hydrocephalus requiring external ventricular drain (**C**)

**Fig. 4:**
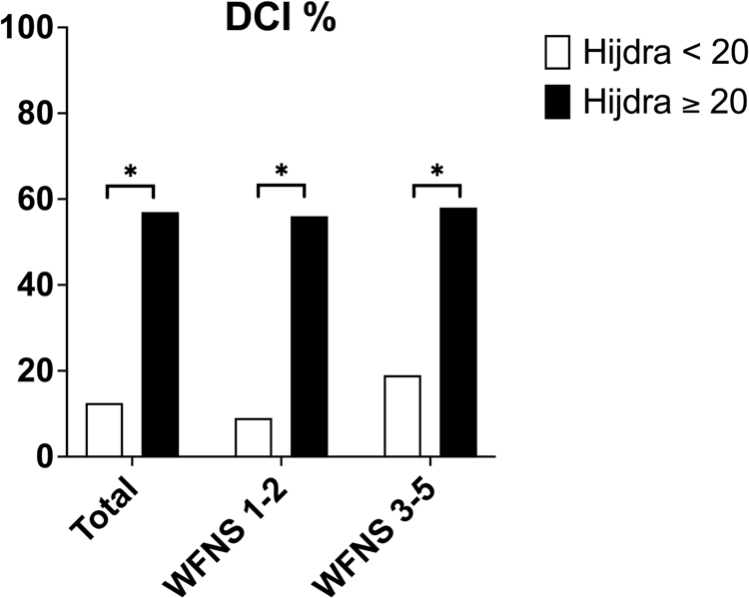
Occurrence of delayed cerebral infarct grouped by Hijdra score < 20 (with bar) and ≥ 20 (black bar) for a total population, low-severity aneurysmal subarachnoid haemorrhage WFNS 1–2 and high-severity aSAH WFNS 3–5 *p < 0.05, Hijdra < 20 vs. Hijdra ≥ 20

For all tested scales or scores, except Fisher grade, a higher value was statistically associated with the occurrence of DCI and poor neurological outcome (Table 2). Figure 3A summarises the results of ROC curves of the main predictors of DCI. Additional file 1—Table S3 shows the cut-off values with corresponding specificity and sensitivity and ROC_AUC_ for each predictor. The Hijdra scale performed best at predicting DCI, with a ROC_AUC_ of 0.80 (95% CI (CI, 0.74–0.85). The ideal cut-off was a Hijdra scale score ≥ 20, with a sensitivity of 85% (95% CI, 75%–92% and a specificity of 63% (95% CI, 54%–71%), but it was less accurate to predict poor outcome (Fig. 3B) than the SAPS II score (ROC_AUC_, respectively, 0.86 (95% CI, 0.71–0.91). Figure 4 shows the occurrence of DCI in the subpopulation of low-severity aSAH (WFNS 1–2) and high-severity aSAH (WFNS 3–5) considering the ideal cut-off score ≥ 20 for the Hijdra scale. In the WFNS 1–2 subpopulation and in the WFNS 3–5 subpopulation the ideal cut-off Hijdra scale score ≥ 20 differentiates statistically significantly between patients with and without DCIs (WFNS 1–2; Hijdra < 20: 9% DCI vs. Hijdra ≥ 20: 56% DCI *p* < 0.05) and (WFNS 3–5 Hijdra < 20: 19% DCI vs. Hijdra ≥ 20: 58% DCI *p* < 0.05). In the WFNS 1–2 subpopulation, the Hijdra scale was also the best-performing scale to predict DCI, with a ROC_AUC_ of 0.82 (95% CI, 0.73–0.89) (Additional file 1—Fig. S1). Cross-analysis outcome—WFNS—HIJDRA is available in the supplemental appendix (Additional file 1—Fig. S2). The IVH score performed best for predicting early hydrocephalus requiring EVD, with a ROC_AUC_ of 0.85 (95% CI, 0.79–0.89) (Fig. 3C). On univariate analysis, the Hijdra scale and IVH score were significant prognostic value for the presence of DCI and of early hydrocephalus requiring EVD (Additional file 1—Table S4).

**Table 2:** Relationship between CT grading systems and the development of poor outcome or delayed cerebral infarct (DCI) in patients with aneurysmal subarachnoid haemorrhage

CT grading systems	All patients (*n* = 230)	Outcome (*n* = 230)			DCI^a^ (*n* = 200)		
		Good (GOSE 5–8) (*n* = 135)	Poor (GOSE 1–4) (*n* = 95)	*p* value^b^	No (*n* = 122)	Yes (*n* = 78)	*p* value^c^
Fisher grade	3 (3)	3 (3)	3 (3)	0.47	3 (3)	3 (3)	0.89
Modified Fisher grade	4 (3, 4)	4 (2–4)	4 (4)	**< 0.001**	4 (2–4)	4 (4)	**< 0.001**
III–IV—*no. (%)*	194 (85)	99 (74)	95 (100)	**< 0.001**	88 (73)	76 (97)	**< 0.001**
Claassen scale	3 (3, 4)	3 (2–4)	4 (3, 4)	**< 0.001**	3 (2–4)	4 (3, 4)	**< 0.001**
III–IV—*no. (%)*	193 (84)	99 (74)	94 (99)	**< 0.001**	88 (73)	76 (97)	**< 0.001**
BNI grading scale	4 (3–5)	4 (3–5)	5 (4, 5)	**< 0.001**	4 (3–5)	4 (4, 5)	**0.003**
IV–V—*no. (%)*	167 (73)	81 (60)	86 (91)	**< 0.001**	73 (60)	67 (86)	**< 0.001**
IVH score	7 (0–11)	5 (0–10)	10 (4–17)	**< 0.001**	5 (0–10)	8 (2–11)	**0.006**
IVH volume—mL	4.1 (1.0–9.0)	2.7 (1.0–7.4)	7.4 (2.0–30.0)	**< 0.001**	2.5 (1.0–7.4)	5.0 (1.5–9.0)	**< 0.001**
Graeb scale	2 (0–5)	2 (0–3)	4 (2–8)	**< 0.001**	1 (0–3)	3 (1–5)	**< 0.001**
Leroux scale	3 (0–7)	2 (0–4)	6 (2–11)	**< 0.001**	2 (0–4)	4 (2–8)	**< 0.001**
Hijdra scale	21 ± 11	18 ± 10	27 ± 9	**< 0.001**	16 ± 10	27 ± 8	**< 0.001**

Results are expressed as numbers (%), medians (interquartile range), or means ± SD

*BNI* Barrow Neurological Institute score, *CT* computerised tomography, *GOSE* Extended Glasgow Outcome Scale, *IVH* intraventricular haemorrhage

^a^ Data for DCI were collected from a collective of 200 patients (30 patients who died before day 7 were excluded)

^b^ *p* value for patients with good outcomes vs. poor outcomes

^c^ *p* value for patients with DCI vs. without DCI. Boldface values represent significant findings assumed at *p* values of 0.05 and below

#### Rating interobserver agreement

All scales were rated as having a good or very good interobserver agreement. The rating scale with the greatest interobserver agreement was the Fisher grade (*κ* = 0.90; 95% CI, 0.77–1.00), followed by the Hijdra score (*κ* = 0.85; 95% CI, 0.80–0.91) (Additional file 1—Table S5). The lowest interobserver agreement was observed with the IVH score (*κ* = 0.69; 95% CI, 0.56–0.83).

#### Multivariable analysis of CT grading systems and clinical parameters

Multivariate statistical analysis of significant risk factors for DCI, poor patient outcome (GOSE 1–4) and early hydrocephalus are presented in Table 3. The Hijdra scale was the only variable with significant prognostic value for the presence of DCI (adjusted odds ratio per unit, 1.18; 95% CI, 1.10–1.25; *p* < 0.001). SAPS II (adjusted odds ratio per unit, 1.06; 95% CI, 1.02–1.10; *p* = 0.002) rebleeding (odds ratio, 9.88; 95% CI, 2.11–46.26; *p* = 0.004), clipping (odds ratio, 2.85; 95% CI, 1.09–7.44; *p* = 0.035) and severe angiospasm (odds ratio, 5.68; 95% CI, 1.73–18.64; *p* = 0.004) were associated with poor outcome. IVH score was the most significant predictor for the occurrence of early hydrocephalus (odds ratio, 1.49; 95% CI, 1.22–1.85; *p* < 0.001). Of interest, SAPS II (adjusted odds ratio per unit, 1.04; 95% CI, 1.00–1.07; *p* = 0.04) and rebleeding (odds ratio, 4.47; 95% CI, 1.12–17.76; *p* = 0.033) were significantly associated with early hydrocephalus occurrence. We constructed a composite score by combining four variables (SAPS2; Neurogenic pulmonary oedema; Hijdra ≥ 20; WFNS). This is the first exploratory step and is presented in additional files (Additional file 1—Table S6).

**Table 3:** Multivariate statistical analysis of significant risk factors for poor patient outcome (GOSE 1–4), delayed cerebral infarct (DCI) and early hydrocephalus requiring external ventricular drainage

	Numerical values^a^	DCI^b^		Poor outcome		Early hydrocephalus	
		Odds ratio (95% CI)	*p* value	Odds ratio (95% CI)	*p* value	Odds ratio (95% CI)	*p* value
SAPS II	0–163	0.99 (0.96–1.02)	0.61	1.06 (1.02–1.10)	**0.002**	1.04 (1.00–1.07)	**0.04**
Clipping	0, 1	0.72 (0.25–2.02)	0.53	2.85 (1.09–7.44)	**0.035**	-	-
Rebleeding	0, 1	0.94 (0.22–3.80)	0.93	9.88 (2.11–46.26)	**0.004**	4.47 (1.12–17.76)	**0.033**
Hijdra scale	0–42	1.18 (1.10–1.25)	**< 0.001**	1.03 (0.97–1.10)	0.38	1.02 (0.96–1.09)	0.47
IVH score	0–23	0.84 (0.70–1.00)	0.06	0.91 (0.75–1.10)	0.32	1.49 (1.22–1.85)	**< 0.001**
Severe angiospasm	0, 1	-	-	5,68 (1.73–18.64)	**0.004**	-	**-**

Boldface values represent significant findings assumed at *p* values of 0.05 and below

The odds ratio needs to be interpreted as follows: for example, for each point increase in *SAPS II*, the risk for poor outcome increases by 6% (95% CI, 3%–10%)

*CI* confidence interval, *IVH* intraventricular haemorrhage, *SAPS II* simplified acute physiologic score II

^a^ 0 = no, 1 = yes

^b^ Data for DCI were collected from a collective of 200 patients (30 patients who died before day 7 were excluded)

## Discussion

Many studies have compared clinical grading scales such as the Hunt and Hess scale, WFNS, and GCS for predicting unfavourable outcomes in aSAH [31, 32]. To our knowledge, however, this study is the first to compare eight radiological scales, grading subarachnoid or ventricular haemorrhage or both for the prediction of DCI, acute hydrocephalus, and functional outcome at 3 months in a large aSAH population. We identified a 39% rate of DCI in our aSAH population, in agreement with studies using a modern definition of DCI [33]. As previously reported, the Fisher grade scale failed to predict vasospasm or DCI occurrence, with both poor sensitivity and poor specificity [6, 7, 9, 10]. We note that in our ICU population, 85% of all patients were classified modified Fisher grade 3–4, which may have confounded statistical analysis.

The Hijdra scale was the most effective scale for predicting DCI, with an ideal cut-off of 20/42 and excellent interobserver agreement. This scale has been studied mostly for its association with functional outcomes and has been found to be superior to the Fisher grade [12, 34]. A German study published in 2023 demonstrates in an older retrospective cohort of 2003–2016, the interest of the HIJDRA scale in the characterisation of the risk of vasospasm in patients with SAH between five other radiological scales [35]. Several aspects differ from our study. In fact, the authors studied an older population covering a large period with a non-actualised definition of DCI. During the period covered by the study, international recommendations were published that could modify local practices for DCI management [25]. In addition, they used the original Fisher score in their comparison, although this score has been replaced by the modified Fisher score due to its non-graduated nature in terms of patient severity [4]. Despite these differences, our results are consistent in terms of the value of the HIJDRA score in the classification of patients with aSAH.

However, another study looked at the value of the Hijdra scale in predicting the presence of an aneurysm in subarachnoid haemorrhage in over 500 patients [36]. The authors report that patients with a HIJDRA score < 22, a WFNS score < 3 and no diffuse meningeal haemorrhage on initial CT scan had a low risk of having an aneurysm on injected CT. According to the authors, this score would enable better patient triage. Dupont et al [37] also reported that a Hijdra score ≥ 23 was strongly associated with the occurrence of vasospasm, and our findings show a strong association of Hijdra score with DCI. In our study, the Hijdra scale did not correlate with the neurological outcome at 3 months. The neurological evolution is not only linked to the occurrence of DCI but also to brain stem lesions generated by intracranial hypertension during SAH ictus, as well as to all the complications that may arise during the long hospitalisation of these patients.

The design of the grading system may explain these results. As DCI development and outcome correlate with the amount of blood on a CT scan [4, 5, 7, 9, 37, 38], grading every cistern and every ventricle likely assesses the overall bleeding with greater precision than other scales. Despite its apparent complexity, the Hijdra scale had good reliability, and we found an excellent interobserver agreement for it [12, 39, 40]. Of interest, both sensitivity and specificity were increased in a WFNS 1–2 subpopulation. In fact, with an ideal cut-off of 18/42, the Hijdra scale had 88% sensitivity and 69% specificity for DCI prognosis (only 4/54 patients had DCI with a Hijdra scale score < 18/42). Associated with clinically predictive factors such as smoking, history of diabetes and hypertension [33], the Hijdra scale could help physicians to better predict DCI occurrence and determine the most appropriate hospitalisation unit for these aSAH patients.

Acute hydrocephalus is a frequent complication after brain aneurysm rupture [41–43]. In our ICU population, 53% of patients needed an EVD. We found that the IVH score best predicted acute hydrocephalus requiring EVD. Moreover, this score stood out from the Graeb and LeRoux scores by allowing for reliable estimation of intraventricular bleeding volume [17]. In a 2012 study, Hwang et al found that the IVH score could reliably predict poor neurological outcome, which was associated with an estimated IVH volume > 6 mL [44]. Recently, the interval to blood clearance in the basal cisterns and peripheral subarachnoid spaces has been associated with shunt dependency. In this study, patients with a shorter interval of blood clearance required a less ventriculo-peritoneal derivation placement than other patients [45]. Thus, accurate evaluation of blood volume on CT scans using quantitative scales or automated computer blood quantification could be useful for aSAH management [46]. The Hijdra scores performed excellently for our primary endpoints. We suggest that, in the future, the scores with confirmed predictive value should be routinely calculated for aSAH patients to either increase the frequency of transcranial Doppler monitoring or to prescribe a systematic CT scan at D7 or also, to improve the timing of preventive measures against impending DCI, such as early initiation of induced hypertension and additional diagnostic confirmation (with digital subtraction angiography or perfusion CT scan) and endovascular treatment of cerebral vasospasm, if present. It could also be used to monitor the lowest-scoring patients in a conventional care setting.

SAPS II was a predictor of poor outcome at 3 and 6 months in our study. This score was generated from a cohort of medical and surgical ICU patients and was not intended to assess mortality in neurological patients. This expected finding illustrates the importance of clinical variables included in the SAPS II scale, particularly age and initial GCS [24]. Indeed, the initial neurological assessment by means of the GCS or WFNS scale is a crucial determinant for neurological outcome and death [23, 31, 47]. New grading scales using both clinical and radiological scores (SAFIR grading scale [48], Southwestern Severity Index [49], PASHPSS [50]) have shown promising results but always use Fisher grade or modified Fisher grade, which is not sufficiently reliable. Future composite scores should include the Hijdra scale to improve accuracy. Because of its complex and time-consuming nature, an automated approach to the Hijdra scale could be developed to facilitate clinical use [46]. Expectedly, we found rebleeding and severe angiospasm as factors of poor neurological outcome in our study. In a recent trial, Stienen et al also showed rebleeding and cerebral infarction as a strong independent predictor of in-hospital mortality with an adjusted odds ratio, respectively, 7.69 and 3.66 [51].

The main limitation of this study arises from its retrospective nature with accordingly lower accuracy and lower completeness of the recorded data, compared to a prospectively collected cohort. Consequently, some degree of bias is inevitable. To avoid some of this inherent bias, the two observers analysing the CT scans were blinded to clinical outcomes and DCI occurrence. Our cohort included neurological ICU patients. Consequently, the less severely ill patients, who probably had the lowest amounts of blood and lowest risk for DCI, were likely not included. The imaging follow-up was not systematically done in the same way for all patients and probably contributed to under-diagnosed complications. However, this represents the real-life management of these patients. Nevertheless, DCI occurred in 39% of patients, which is comparable to proportions in other recent studies [33].

## Conclusion

Radiological grading of SAH is useful for predicting DCI risk. Among these scales, the Hijdra scale seems to be the most effective at predicting the occurrence of DCI. An automated computer quantification approach for this scale could facilitate its daily use. This evaluation associated with clinical predictive values could help intensivists and neurosurgeons better use critical care resources for these patients.

## Supplementary information

Electronic Supplementary Material

## Abbreviations

aSAH: Aneurysmal subarachnoid haemorrhage
CI: Confidence interval
DCI: Delayed cerebral infarct
EVD: External ventricular drainage
GCS: Glasgow Coma Scale
GOSE: Glasgow Outcome Scale Extended
ICU: Intensive care unit
IVH: Intraventricular haemorrhage
NCCU: Neurocritical care unit
ROC: Receiver operating characteristic
SAPS II: Simplified Acute Physiology Score 2
WFNS: World Federation of Neurosurgical Societies
