# Supplementary material for: Comparison of scales for the evaluation of aneurysmal subarachnoid haemorrhage: a retrospective cohort study
Source: Eur Radiol. 2024 Jun 5;34(11):7526–36. doi: 10.1007/s00330-024-10814-4 (PMC11519170; doi:10.1007/s00330-024-10814-4)
Supplement: Supplementary file 1 — Electronic Supplementary Material [file 330_2024_10814_MOESM1_ESM.pdf]

Comparison of scales for the evaluation of aneurysmal subarachnoid haemorrhage: a retrospective cohort study

Electronic Supplementary Material (ESM)

TABLE OF CONTENTS

Figure S1. Receiver operating characteristic curves of Hijdra scale in WFNS 1-2 patients sub-population used for determining the development of delayed cerebral ischemia. 3

Table S1. Comparison of CT-scan based grading systems for subarachnoid hemorrhage and intraventricular hemorrhage 4

Table S2. Univariate risk factors for death 5

Table S3. Area under the receiver operating characteristic curve (ROC_{AUC}) values and cut-off values to determine occurrence of delayed cerebral ischemia (DCI), early hydrocephalus requiring external ventricular drainage and poor patient outcome (GOSE 1-4) after aneurysmal subarachnoid hemorrhage 6

Figure S2. Cross-analysis Outcome – WFNS – HIJDRA 8

Table S4. Univariate risk factors for poor patient outcome (GOSE 1-4), delayed cerebral ischemia (DCI) and early hydrocephalus requiring external ventricular drainage. 9

Table S5. Comparison of Kappa Values (κ) between Observers for the Different Qualitative Scales and Quartile Distribution of Semi quantitative and Quantitative Values 11

Table S6. Composite score..

Figure S1. Receiver operating characteristic curves of Hijdra scale in WFNS 1-2 patients sub-population used for determining the development of delayed cerebral ischemia. Area under the receiver operating characteristic curve at 0.82 (95% CI, 0.73 to 0.89). The ideal cut-off was an Hijdra scale ≥ 18 with a sensitivity of 87.8% (95% CI, 72 to 97) and a specificity of 69% (95% CI, 57 to 80).

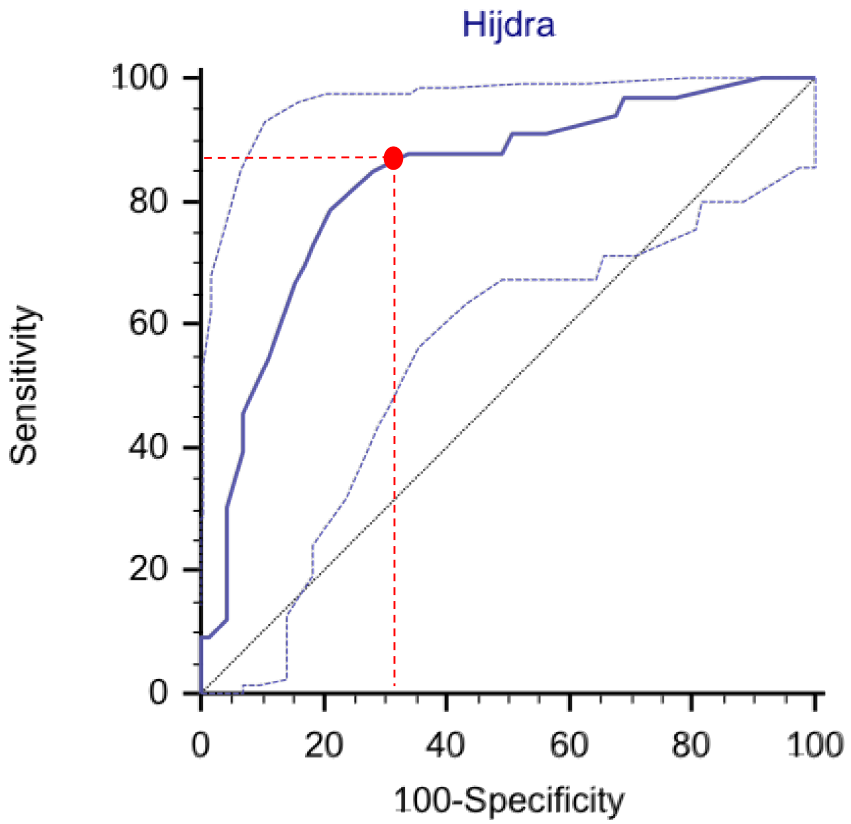

Table S1. Comparison of CT-Scan based grading systems for subarachnoid hemorrhage and intraventricular hemorrhage.

Grading systems (*range*)	Fisher grade (1-4)	modified Fisher grade † (0-4)	Claassen scale † (0-4)	BNI scale † (1-5)	Graeb scale (0-12)		LeRoux scale (0-16)	Hijdra scale (0-42)		IVH score (0-23)	
					**For each lateral ventricle**	**For 3rd and 4th ventricles**	**For each ventricle**	**Sub-arachnoid compartment †** *(for each cistern or fissure)*	**Ventricular compartment ‡** *(for each ventricle)*	**For each lateral ventricle**	**For 3rd and 4th ventricles**
0	-	No blood	No blood	-	No blood	No blood	No blood	No blood	No blood	No blood or small amount of layering	No blood
1	No Blood	Thin SAH, no IVH	Thin SAH, no IVH in either lateral ventricle	No Blood	Trace of blood	Blood present, size normal	Trace of blood	Small amount of blood	Blood sedimentation in the posterior part	Up to 1/3 filled with blood	Partially or completely filled
2	SAH <1mm	Thin SAH with IVH	Thin SAH, IVH in both lateral ventricles	≤5mm	< 50% filled	Filled with blood and expanded	< 50% filled with blood	Moderately filled with blood	Partly filled with blood	Up to 2/3 filled with blood	-
3	SAH ≥1mm	Thick SAH, no IVH	Thick SAH, no IVH in both lateral Ventricles	>5mm and ≤10mm	> 50% filled	-	> 50% filled of blood	Completely filled with blood	Completely filled with blood	Mostly or completely filled with blood	-
4	Diffuse or no SAH with IVH	Thick SAH with IVH	Thick SAH, IVH in both lateral ventricles	>10mm and ≤15mm	Completely filled and expanded	-	Completely filled and expanded	-	-	-	-
5	-	-	-	>15mm	-	-	-	-	-	-	-

modified Fisher grade: † Definition of thin or thick SAH are not specified

Claassen scale: † Thick SAH is defined by a thickness of the blood clot ≥5mm completely filing a cistern or fissure.

BNI scale: † Maximum thickness of the blood clot measured perpendicular to the most predominantly involved cistern or fissure.

Hijdra scale: † Ten cisterns are analysed: frontal interhemispheric (A); lateral sylvian fissure (two each; B), medial sylvian fissure (two each; C), suprasellar (two each; D), and ambient (two each; E); and quadrigeminal (F). Therefore, the total amount of subarachnoid blood ranges from 0 to 30 points on the scale.

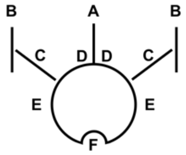

‡ The four ventricles are analyzed. Therefore, the total amount of blood within the four ventricles ranges from 0 to 12 points on the scale.

IVH score: † IVHS = 3 x (RV + LV + H) + III + IV
where RV stands for right ventricle score, LV for left vent
for third ventricle score, IV for fourth ventricle score, and
presence of hydrocephalus.
The formula for converting IVHS to volume is :
IVH volume (mL) = $e^{IVHS/5}$

BNI denotes Barrow Neurological Institute score; IVH, intraventricular haemorrhage; SAH, subarachnoid haemorrhage

Table S2: Univariate risk factors for death

	OddRatio	IC25	IC95	*P* value
**Age**	1,03	0,99	1,07	0,178
**IGS2**	1,08	1,04	1,13	**0,000**
**WFNS**	0,73	0,34	1,51	0,397
**GSW**	0,84	0,66	1,06	0,145
**Surgery**	2,44	0,87	6,95	0,091
**HIP**	1,02	0,33	3,18	0,973
**Rebleed**	6,82	1,84	27,52	**0,005**
**Hijdra**	1,07	0,99	1,16	0,123
**HIVScore**	1,01	0,84	1,19	0,951
**BNI**	1,30	0,59	2,89	0,518
**MCA**	0,80	0,25	2,44	0,697
**OAP**	0,21	0,01	1,68	0,203
**Leroux**	1,09	0,70	1,67	0,707
**Graeb**	0,90	0,46	1,77	0,750
**FisherO**	0,75	0,12	5,29	0,762
**FisherM**	1,19	0,23	6,22	0,833
**Claassen**	0,84	0,24	2,90	0,782

Boldface values represent significant findings assumed at P-values of .05 and below

Table S3. Area under the receiver operating characteristic curve (ROC_{AUC}) values and cut-off values to determine occurrence of delayed cerebral ischemia (DCI), early hydrocephalus requiring external ventricular drainage and poor patient outcome (GOSE 1-4) after aneurysmal subarachnoid hemorrhage. *

Variables	ROC_{AUC} *(95% confidence interval)*	Optimal Cut-off	Specificity	Sensitivity
			Expressed in percent (95% confidence interval)	
DCI				
Fisher Grade	0.52 (0.47–0.59) #	≥2	98 (92–99)	18 (11–27)
modified Fisher Grade	0.67 (0.60–0.73) #	≥3 -	50 (41–59)	79 (69–88)
Claassen Scale	0.66 (0.59–0.72) #	≥2	27 (19–36)	97 (91–99)
BNI Scale	0.63 (0.56–0.70) #	≥3 -	40 (31–49)	86 (76–93)
Hijdra Scale	0.80 (0.74–0.85)	≥20	63 (54–71)	85 (75–92)
LeRoux Scale	0.65 (0.58–0.71) #	≥4	78 (70–85)	47 (36–59)
Graeb Scale	0.64 (0.57–0.71) #	≥1	51 (42–60)	73 (62–83)
Intraventricular Haemorrhage Volume Score	0.61 (0.54–0.68) #	≥1	40 (31–50)	82 (72–90)
GCS score	0.63 (0.56–0.69) #	≤14	40 (31–49)	83 (73–91)
WFNS Scale	0.61 (0.53–0.67) #	≥1	40 (31–49)	81 (70–89)
SAPS II	0.56 (0.48–0.63) #	≥41	75 (67–82)	37 (25–50)
Early hydrocephalus requiring EVD				
Fisher Grade	0.57 (0.51–0.64) †	≥2	17 (11–26)	99 (95–100)
modified Fisher Grade	0.72 (0.66–0.78) †	≥3	57 (47–66)	87 (79–92)
Claassen Scale	0.72 (0.66–0.78) †	≥3	76 (67–84)	64 (55–73)
BNI Scale	0.61 (0.55–0.68) †	≥3	36 (27–46)	80 (72–87)
Hijdra Scale	0.75 (0.69–0.80) †	≥26	82 (73–88)	59 (49–68)
LeRoux Scale	0.80 (0.75–0.85) †	≥2	69 (59–77)	79 (71–86)
Graeb Scale	0.82 (0.76–0.86) †	≥2	78 (69–85)	73 (64–80)
Intraventricular Haemorrhage Volume Score	0.85 (0.79–0.89)	≥8	90 (83–95)	70 (31–78)
GCS score	0.70 (0.66–0.78) †	≤12	73 (64–81)	64 (55–73)
WFNS Scale	0.68 (0.61–0.74) †	≥3	73 (64–81)	63 (54–71)
SAPS II	0.73 (0.66–0.78) †	≥28	67 (57–78)	77 (68–84)
Poor outcome				
Fisher Grade	0.52 (0.45–0.59) §	≥2	14 (9–22)	100 (96–100)
modified Fisher Grade	0.66 (0.60–0.72) §	≥3 -	45 (37–54)	83 (74–90)
Claassen Scale	0.71 (0.64–0.76) §	≥3	68 (59–76)	65 (54–74)
BNI Scale	0.73 (0.67–0.78) §	≥4 -	73 (65–80)	65 (54–74)
Hijdra Scale	0.73 (0.67–0.79) §	≥23	66 (57–74)	72 (62–81)
LeRoux Scale	0.70 (0.63–0.76) §	≥4	78 (70–85)	60 (50–70)
Graeb Scale	0.70 (0.63–0.76) §	≥3	76 (68–83)	58 (47–68)
Intraventricular Haemorrhage Volume Score	0.68 (0.62–0.74) §	≥7 -	66 (57–74)	65 (54–74)

GCS score	0.79 (0.73–0.84)	≤12	75 (67–82)	77 (67–85)
WFNS Scale	0.79 (0.74–0.84)	≥2	70 (61–77)	83 (74–90)
SAPS II	0.83 (0.77–0.88)	≥33	72 (63–79)	83 (74–90)

* Data for DCI were collected from a collective of 200 patients (30 patients who died before day 7 were excluded).
Data for poor outcome, early hydrocephalus requiring EVD and mortality were collected from a collective of 230 patients.
# ROC_{AUC} significantly different than the one of the Hijdra scale ($p<0.05$). † ROC_{AUC} significantly different than the one of the Intraventricular Haemorrhage Volume Score ($P<0.05$).
§ ROC_{AUC} significantly different than the one of the SAPS II scale ($p<0.05$).
BNI denotes average Barrow Neurological Institute; DCI, Delayed Cerebral Infarction; EVD, External Ventricular Drain; GCS, Glasgow Coma Scale; SAH, Subarachnoid Haemorrhage; SAPS, Simplified Acute Physiology Score and WFNS, World Federation of Neurological Surgeons Grading System.

Figure S2: Cross-analysis Outcome – WFNS – HIJDRA.

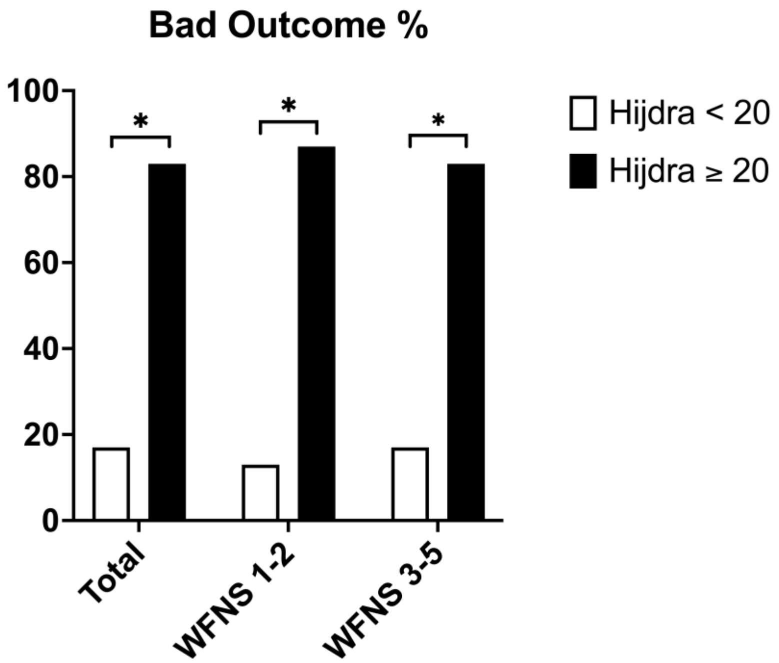

There was a significant difference in the percentage of bad outcome in both the total population and the WFNS 1-2 and 3-5 subgroups according to cut-off 20 of the HIJDRA scale. * p<0.05

Table S4. Univariate risk factors for poor patient outcome (GOSE 1-4), delayed cerebral ischemia (DCI) and early hydrocephalus requiring external ventricular drainage.

	Numerical values*	Poor outcome		DCI†		Early hydrocephalus	
		Odds ratio [95% CI]	*P* value	Odds ratio [95% CI]	*P* value	Odds ratio [95% CI]	*P* value
Clinical characteristics							
Age	18-87	1.0 [1.0-1.1]	**0.003**	1.0 [1.0-1.0]	0.20	1.0 [1.0-1.0]	0.51
Male sex	0, 1	1.8 [1.0-3.3]	0.06	1.2 [0.7-2.1]	0.59	1.3 [0.8-2.2]	0.35
SAPS II	0-163	1.1 [1.1-1.1]	**<0.001**	1.0 [1.0-1.0]	0.12	1.1 [1.0-1.1]	**0.05**
WFNS scale	1-5	2.3 [1.8-3.1]	**<0.001**	1.2 [1.0-1.5]	**0.02**	1.6 [1.3-1.9]	**<0.001**
GCS	3-15	0.8 [0.7-0.8]	**<0.001**	0.9 [0.9-1.0]	**0.03**	0.8 [0.8-0.9]	**<0.001**
Aneurysm Clipping	0, 1	4.2 [2.2 -8.0]	**<0.001**	0.6 [0.3-1.3]	0.22	0.8 [0.4-1.4]	0.43
Neurogenic pulmonary oedema	0, 1	0.6 [0.1 -2.2]	0.46	6 [1.6-46]	**0.02**	1.4 [0.4-5.5]	0.63
CT scan findings							
Intracerebral hematoma	0, 1	2.5 [1.3 -4.7]	**0.005**	0.8 [0.4-1.6]	0.55	1.3 [0.7-2.2]	0.44
Rebleeding	0, 1	4.1 [1.6 -10.5]	**0.003**	0.8 [0.3-2.3]	0.74	2.4 [0.9-7.0]	0.08
Ruptured aneurysm MCA	0, 1	2.4 [1.3-2.6]	**<0.001**	0.7 [0.4-1.5]	0.45	0.6 [0.3-1.2]	0.16
Fisher grade	1-4	1.2 [0.6-2.5]	0.56	1.2 [0.5-2.6]	0.70	3.3 [1.6-7.9]	**0.003**
modified Fisher grade	0-4	3.5 [1.5-7.9]	**0.001**	2.7 [1.8-4.4]	**<0.001**	3.0 [2.1-4.7]	**<0.001**
Claassen scale	0-4	2.8 [1.7-4.8]	**<0.001**	2.4 [1.6-3.6]	**<0.001**	2.7 [1.9-3.9]	**<0.001**
BNI grading scale	1-5	2.9 [2.0-4.2]	**<0.001**	1.8 [1.0-3.3]	**0.001**	1.6 [1.2-2.1]	**<0.001**
Hijdra scale	0-42	1.1 [1.1-1.2]	**<0.001**	1.1 [1.1-1.2]	**<0.001**	1.1 [1.1-1.1]	**<0.001**
Graeb scale	0-12	1.2 [1.1-1.3]	**<0.001**	1.1 [1.0-1.2]	**0.01**	1.5 [1.3-1.7]	**<0.001**
LeRoux scale	0-16	1.2 [1.1-1.2]	**<0.001**	1.1 [1.1-1.2]	**0.008**	1.3 [1.0-1.1]	**<0.001**
IVH score	0-23	1.1 [1.1-1.2]	**<0.001**	1.0 [1.0-1.1]	**0.04**	1.3 [1.2-1.4]	**<0.001**

* 0=no, 1=yes. †Data for DCI were collected from a collective of 200 patients (30 patients who died before day 7 were excluded).
Boldface values represent significant findings assumed at P-values of .05 and below.
The odds ratio needs to be interpreted as follows: for example, each point increase in Hijdra scale, the risk for DCI increases by 10% (95% CI, 3%–10%).
CI denotes Confidence Interval; BNI, Barrow Neurological Institute score; GCS, Glasgow Coma Scale; GOSE, Extended Glasgow Outcome; IVH, Intraventricular Haemorrhage; SAPS II, Simplified Acute Physiologic Score II and WFNS, World Federation of Neurological Surgeons Grading System.

Table S5. Comparison of Kappa Values (κ) between observers for the different qualitative scales and quartile distribution of semi quantitative and quantitative values.

**Grading systems**	**κ value**	***95% confidence interval***	**Strength of agreement**
Fisher Scale	0.90	0.77–1.00	Very good
Fisher modified Scale	0.80	0.57–1.00	Good
Claassen Scale	0.70	0.48–0.92	Good
BNI Scale	0.72	0.54–0.90	Good
Hijdra Scale	0.85	0.80–0.91	Very good
LeRoux Score	0.84	0.74–0.94	Very good
Graeb Score	0.85	0.75–0.94	Very good
IVH Score	0.69	0.56–0.83	Good

BNI denotes average Barrow Neurological Institute and IVH; Intraventricular Hemorrhage.

Table S6. Composite score

Score formula: DCI score=-SAPS2+238*OAP_Neuro+286*HijdraSup20+8WFNS

	Points	
	0	1
**Hijdra≥20**	0	286
**SAPS22**	-1 X Score IGS2	
**pulmonary_oedema1**	0	238
**WFNS**	8 X score WFNS	

Results

AUC	lower	upper	sensitivity	specificity
76	69,4	82,6	0,9359	0,5738
